# The Use of a New CellCollector to Isolate Circulating Tumor Cells from the Blood of Patients with Different Stages of Prostate Cancer and Clinical Outcomes - A Proof-of-Concept Study

**DOI:** 10.1371/journal.pone.0158354

**Published:** 2016-08-01

**Authors:** Gerit Theil, Kersten Fischer, Ekkehard Weber, Rita Medek, Raschid Hoda, Klaus Lücke, Paolo Fornara

**Affiliations:** 1 Martin Luther University Halle-Wittenberg, Clinic of Urology and Transplantation, Ernst-Grube-Str.40, 06120, Halle/Saale, Germany; 2 GILUPI-GmbH, Potsdam, Mühlenberg 11, Germany; 3 Martin Luther University Halle-Wittenberg, Institute of Physiological Chemistry, Hollystr. 1, 06120, Halle/Saale, Germany; National Health Research Institutes, TAIWAN

## Abstract

**Background and Methods:**

Circulating tumor cells (CTCs) constitute a useful approach for personalized medicine. Nevertheless, the isolation of these cells remains very challenging because they rarely circulate in the blood. Another current problem is the cancer-specific characterization of these cells, which requires a method that allows for the molecular and immunocytochemical profiling of all captured cells. The purpose of our proof of concept study was to investigate the use of a medical wire (CellCollector, GILUPI) to isolate CTCs in the blood of prostate cancer (PCa) patients, which allowed CTCs to be counted and molecularly characterized. Forty-three PCa patients in different stages and 11 control subjects were studied. Some randomized samples were used to detect tumor-associated transcripts, such as prostate-specific membrane antigen (PSMA), prostate-specific antigen (PSA) and epidermal growth factor receptor (EGFR), in the isolated CTCs.

**Results:**

The mean CTC counts were 4.6 CTCs [range, 0–8] in patients with localized PCa, 16.8 CTCs [range, 10–25] in patients with locally advanced PCa, and 26.8 CTCs [range, 0–98] in patients with metastatic PCa. The median follow-up time was 24 months, and there was a significant difference in the cancer-specific survival rates. Patients with CTC counts under 5 CTCs lived significantly longer (p = 0.035) than patients with more than 5 CTCs. We also demonstrated that the captured CTCs could be molecularly characterized. We detected tumor-associated transcripts of EGFR and PSMA in patients with metastatic PCa in 42.8% and 14.3% of the analyzed samples, respectively.

**Conclusion:**

Our results indicate that the sensitive isolation and molecular characterization of CTCs can be achieved ex vivo using the wire. Patients with more than 5 CTCs had a mortality risk that was 7.0 times greater that of those with fewer than 5 CTCs (hazard ratio 7.0 95%, CI 1.1–29.39). This proof of concept was required for the approval of the use of the CellCollector in a clinical study for the in vivo isolation of CTCs from the blood stream of PCa patients by the Federal Institute for Drugs and Medical devices (Germany, BfArM).

## Introduction

Prostate cancer (PCa) is characterized by heterogeneous phenotypes that display a broad range of clinical outcomes from relatively indolent to lethal metastatic disease. The standard diagnostic tools include the level of prostate-specific antigen (PSA) in the blood, transrectal ultrasonography (TRUS), histological Gleason grading of biopsy specimens, and clinical tumor, node, metastasis (TNM) staging, which are all insufficient for an accurate risk stratification of each patient. Given the wide range of clinical outcomes and the heterogeneity of the disease, physicians’ main challenge remains distinguishing latent tumors from clinically significant ones. Thus, there is a clear need for additional, improved prognostic markers [1]. Circulating tumor cells (CTCs) might represent a new opportunity for the management and monitoring of disease progression. Recent clinical trials have demonstrated that the presence of CTCs in metastatic breast, prostate, and colorectal cancer patients are frequently proportional to survival [2–4]. The clinical usefulness of these cells include the prediction of clinical tumors, disease prognosis, therapeutic monitoring, and therapeutic outcome. However, they have not been used in the clinical setting.

The most important challenge facing CTC research is the sensitive isolation (enrichment) of these rare cells, which typically present as a single tumor cell against a background of millions of blood cells [5]. Although many approaches for detecting and characterizing CTCs from blood samples exist, the only standardized CTC-isolation platform is the CellSearch^®^ System (Janssen Diagnostics), which has been cleared by the US Food and Drug Administration (FDA) for monitoring metastatic breast, colon and prostate cancers [2,3,6]. This isolation technique is based on the use of magnetic beads coated with antibodies against the epithelial cell adhesion molecule (EpCAM) to capture EpCAM-expressing cells, followed by immunostaining of the captured cells. The cell-enumeration results are always expressed as the number of CTCs per 7.5 ml of blood [7]. The limitation of all current systems is the small blood volume available for CTC enrichment, which is related to the relatively low sensitivity of this approach [8].

Here, we investigated the use of the new CellCollector, which is usually used for the *in vivo* isolation of CTCs. Previously, the wire was only applied *in vivo* through a standard venous cannula into the cubital veins of 12 breast and 12 non-small lung cancer patients, and CTCs were detected in all examined tumor stages, including early-stage cancer [9]. We used the CellCollector to capture EpCAM-positive cells in a very small number of PCa patients to demonstrate the general feasibility of capturing CTCs for this type of cancer.

This proof-of-concept study was urgently needed because the wire could not be used *in vivo* in prostate cancer patients. Therefore, we determined whether the CellCollector could be used *ex vivo* for the isolation of CTCs from blood samples (15 ml) obtained from PCa patients with localized, locally advanced and metastatic cancer and a control group. Captured CTCs were identified based on the intensity of the cytokeratin immunofluorescence signal and the location of Hoechst 33258 nuclear stained cells. The cytomorphological and immunofluorescence staining parameters are identical to those of the FDA cleared CellSearch system.

**Fig 1:**
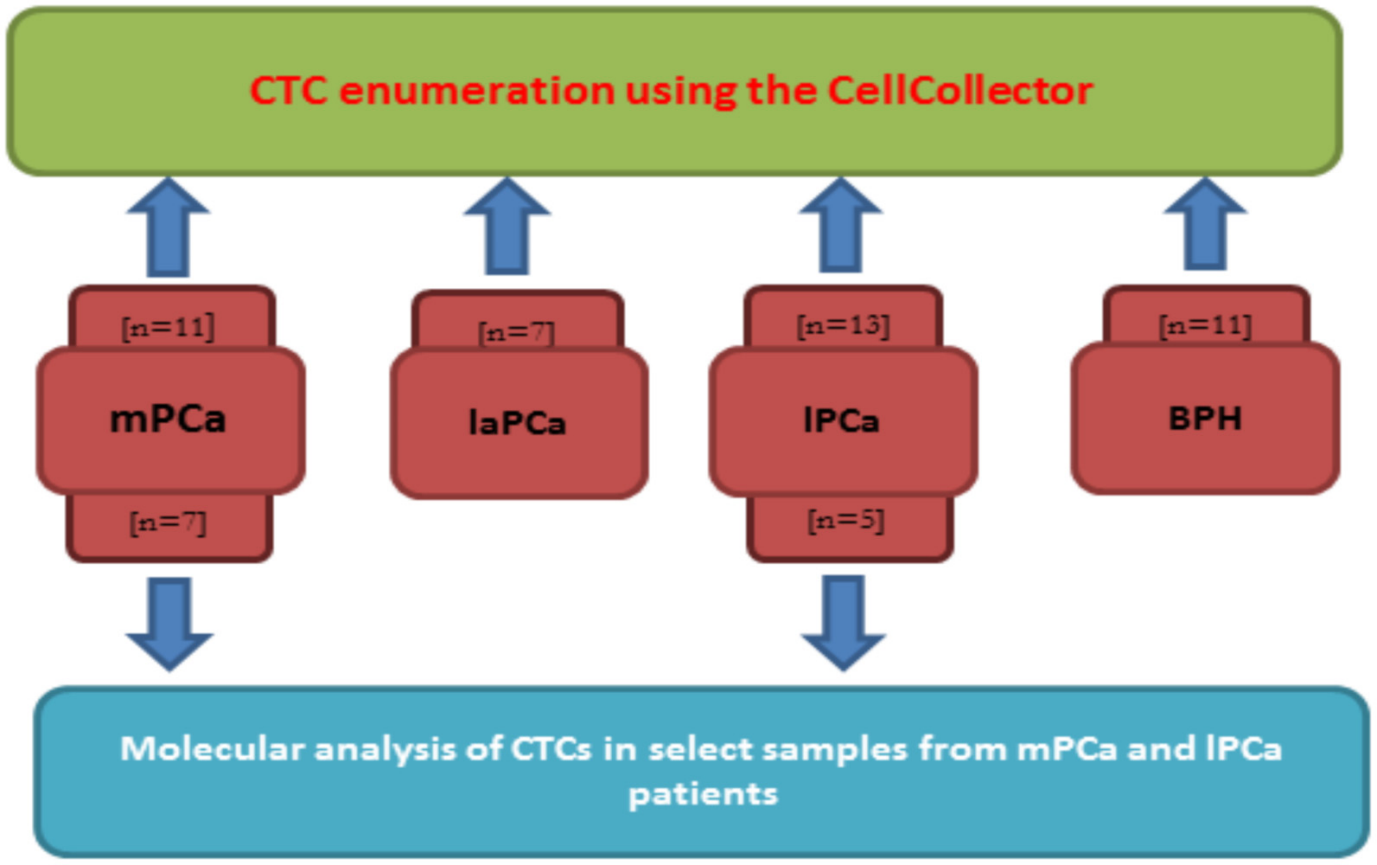
Study design (n = number of blood samples). Localized Prostate Cancer (lPCa), locally advanced Prostate Cancer (laPCa), metastatic Prostate Cancer (mPCa).

Additionally, we investigated the application of a molecular analysis technique to the CTCs captured by the wire tip (Fig 1). Using this technique, it was possible to obtain information regarding modifications in intracellular signaling events in response to therapy. This information is urgently needed for the broad implementation of CTCs in clinical practices.

## Patients and Methods

### Patients and blood sample collection

Our preliminary study was approved by the local ethics committee of Martin Luther University Medical School Halle. Written, informed consent was obtained from all patients and volunteers. Patients were enrolled from our clinic from May 2009 to August 2010. These patients were classified into three groups: the first group had localized prostate cancer (lPCa; stage T1-T2 NO, M0, R1), the second group had locally advanced PCa (laPCa; stage T3-T4 N0, M0, Rx), and the third group consisted of patients with metastatic PCa (mPCa; stage T4 N1-N3/M1). Patients in the mPCa group were required to have documented, computed tomography (CT)-confirmed metastases. The control group consisted of volunteers with benign prostatic hyperplasia (BPH) and no evidence of PCa. Patients with second malignancies were excluded.

The samples were stored for a maximum of 4 h at room temperature before processing. Samples of blood (15 ml) were drawn into ethylenediaminetetraacetic acid (EDTA) tubes, and 4 ml of serum was used for the determination of PSA.

### Antibody functionalization of the CellCollector

The CellCollector is a 16-cm-long stainless steel wire commonly used in medicine. The interaction of target CTCs with the wire is mediated by an antibody (chiHEA 125, GILUPI) directed against EpCAM, which is overexpressed in different cancer cell types. This antibody is regularly used in humans.

**Fig 2:**
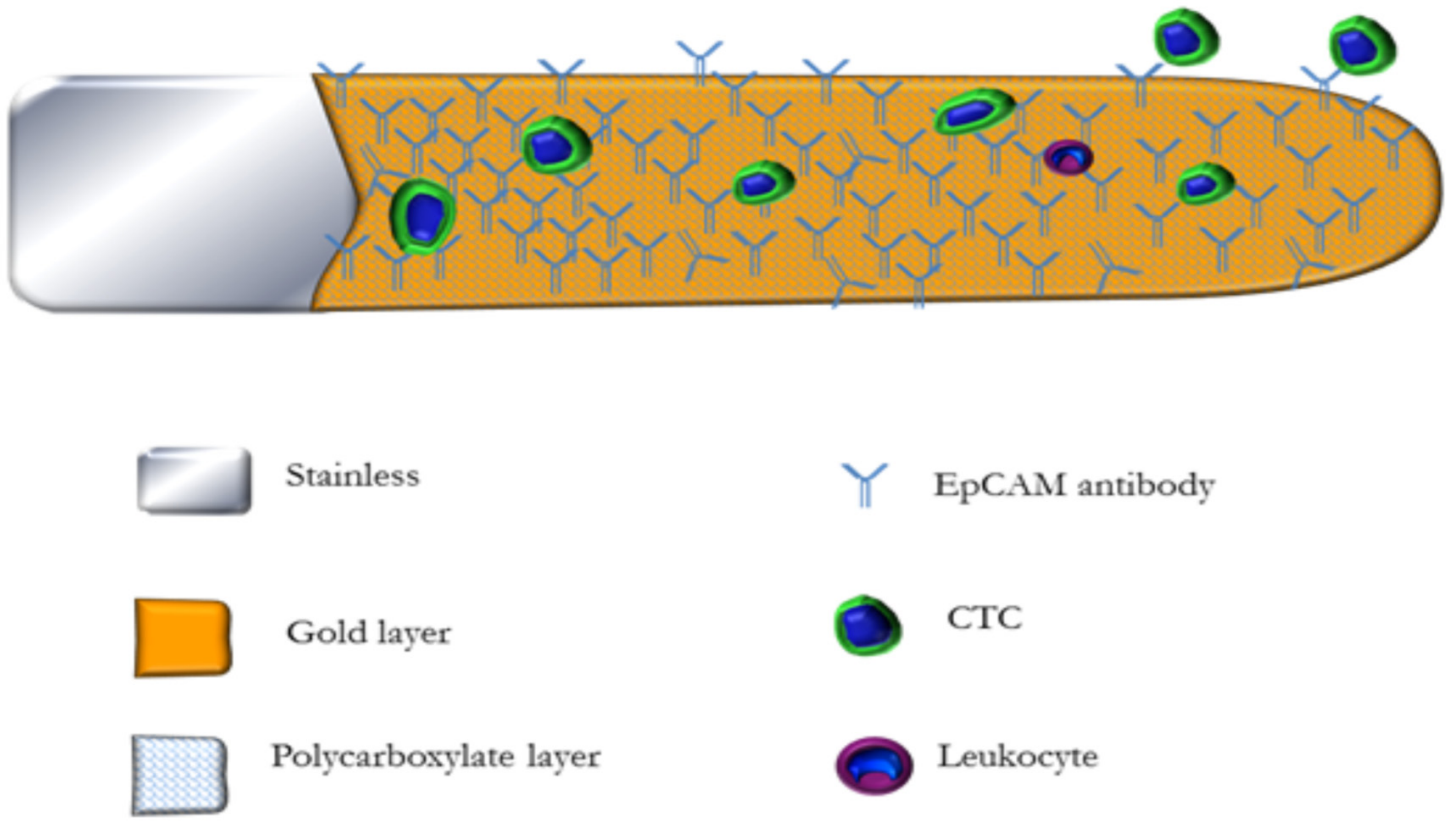
Structural details of the CellCollector.

The tip of the wire was functionalized: a 2-cm portion of the wire was coated with a 0.2-μm-thick layer of gold covered by a 1-5-μm polycarboxylate layer (Fig 2). The functionalization of the wire began with the rehydration of the hydrogel by incubating the wire in sterile distilled water for 15 min. The layer was then activated by incubation in a 1-ethyl-3-(3-dimethylaminopropyl) carbodiimide hydrochloride/N-hydroxysulfosuccinimide (EDC/NHS) solution (Sigma) at 22°C for 20 min. NHS (100 mM) was prepared in 50-mM 2-(N-morpholino)ethanesulfonic acid (MES) buffer (Sigma) at pH 5.3. Then, 0.5% of EDC (Sigma) was added to the solution. After activation, the wire tip was rinsed with 5-mM acetic acid (Roth) and functionalized by incubating it with the anti-EpCAM antibody for 1 h at 22°C. The hydrogel was covalently coupled to the anti-EpCAM antibody. Free carboxyl groups on the hydrogel were blocked with 1-M ethanolaminehydrochloride (Sigma) at pH 8.5 (30 min, room temperature), and unbound antibodies were removed from the wire by washing three times with distilled water [9]. The functionalized wires were stored in distilled water at 4°C until use.

### Microfluidic capture of CTCs

This method was first established by experiments with blood that was enriched with cells of the EpCAM-positive PCa cell line LNCapLNCap [10,11], and the performance of the CellCollector was demonstrated with a hemodynamic flow system. The LNCap cells were harvested by trypsin EDTA treatment. To determine cell numbers, an 10 μl-aliquot of cells was placed on a hemocytometer plate, and counts were performed using a conventional inverted microscope.

The parameters of the dynamic model were adapted to the hemodynamic parameters of the peripheral venous blood circulation. The velocity of the blood in the flow system’s chamber (d = 3 mm) was 1.2 cm/s, and the flow speed in a medium-sized human vein is 1–5 cm/s. The functionalized anti-EpCAM wire was inserted into the flow chamber through a septum via a cannula. The flow system was filled with 15 ml of blood, and the blood was pumped through the flow chamber for 14 cycles (30 min). Patient blood samples were subjected to the same conditions.

### Immunocytochemical analysis and CTC enumeration

CTCs were identified by immunocytochemical staining and enumerated by a blinded, trained operator. After being incubated with blood in the flow system, the wire was washed three times in phosphate-buffered saline (PBS) (Sigma). Bound cells were fixed with 4% paraformaldehyde (Sigma) for 10 min at room temperature. Subsequently, the cells were blocked with 3% bovine serum albumin (BSA) (PAA) in PBS for 1 h. Primary antibodies, including anti-pan-cytokeratin-fluorescein isothiocyanate (FITC) (CK8, CK18 and CK19, Abcam) and anti-CD45- allophycocyanin (APC, Invitrogen), were added for 1 h. The wire was then rinsed 3 times with 3 ml of PBS, and the nuclei were counterstained with Hoechst 33258 (Invitrogen). CTCs were identified using a Nikon Eclipse E600 fluorescent microscope with a 20x objective. Fluorescent images were recorded with a Vosskühler CCD-1300-QLN-Camera. The images were digitally processed with ImageJ software by altering contrast and brightness in accordance with the Nature Publishing Guidelines. A cell was considered to be a CTC if it was positively stained for pan-cytokeratin, it was negative for CD45, and certain morphological criteria for tumor cells were met: the presence of a nucleus with a round or ellipsoid shape and a cell size ranging from 5 to 50 μm. Leucocytes were defined as nucleated (Hoechst-positive), CD45-positive and pan-cytokeratin-negative cells.

### Identification of tumor-specific transcripts in CTCs

First, we performed tumor cell spiking experiments with the PCa cell line LNCap. We enriched EDTA blood samples from healthy donors with a defined cell count (50, 200, or 500 LNCap cells/15 ml blood). Subsequently, the wires were incubated with prepared blood samples in the microfluidic chamber under the conditions described above. The next step was the analysis of 7 blood samples of metastatic PCa patients and 5 localized PCa patients.

After the incubation, the wire was rinsed 3 times with PBS. To isolate mRNA from the captured cells, the functionalized tip of the wire (2 cm) was cut into 5-mm pieces and incubated for 1 min in 100 μl of lysis buffer. The lysis buffer and primers used were components of the commercially available AdnaTest ProstateCancerDetect kit (Adnagen) [12]. Subsequently, the mRNA from lysed cells was recovered by magnetic separation using Oligo (dT) 25 Dynabeads^®^. The mRNA/bead complex was reverse transcribed using Sensiscript^™^ Reverse Transcriptase (Qiagen) and RNase inhibitor (Promega). The reverse transcription into cDNA was performed in a one-step reaction (60 min at 37°C min and 5 min at 95°C). The samples were cooled at 4°C and stored at -20°C. The tumor-associated mRNA transcripts from the captured CTCs were analyzed using a multiplex polymerase chain reaction (PCR). The primer mixture allowed for the amplification of three tumor-associated antigens prostate-specific membrane antigen (PSMA), PSA, and epidermal growth factor receptor (EGFR) and the housekeeping gene actin. The primers generated fragments of the following sizes: PSMA, 449 base pairs (bp); PSA, 357 bp; EGFR 163, bp; and actin, 120 bp. In the negative controls, the mRNA and cDNA were replaced with water in the reverse transcription and PCR experiments. The samples were evaluated using the BioAnalyzer 2100 (Agilent Technologies, Santa Clara, CA, USA) on a DNA 1000 LabChip.

The test was considered CTC-positive for PCa if a PCR fragment of at least one tumor-associated transcript (PSA, PSMA or EGFR) and a fragment of the control gene were clearly detected. The peaks with a concentration of ≥ 0.10 ng/μl were considered positive.

### Cell lines and culture conditions

The prostatic human tumor cell line LNCap (ATCC), which was established from tumor tissue removed from a metastatic lesion of a man with a prostate carcinoma, was cultured in RPMI 1640 (Invitrogen) medium supplemented with 10% inactivated fetal calf serum (FCS), 2-mM L-glutamine, and 80 g/l gentamycin in a 5% CO_2_ humidified incubator at 37°C [10,11].

### Statistical design

Statistical analyses were performed and Figs generated using Prism 6 (GraphPad Software, La Jolla, CA). A *p*-value of ≤ 0.05 was considered significant. CTCs were correlated with the PSA level and Gleason sum by determining the Spearman rank correlation coefficients (r). The time-to-event outcome (overall survival [OS]) was evaluated using Kaplan-Meier methods, and the survival-time differences were compared using the log-rank (Mantel-Cox) test.

## Results

### Patient characteristics

We enrolled 43 PCa patients, and 11 volunteers with BPH served as the control group. Table 1 shows the clinicopathological characteristics of patients with localized PCa (n = 18), locally advanced PCa (n = 7), and metastatic PCa (n = 18). The control group included 11 patients with histopathologically confirmed BPH and no evidence of PCa. The median ages were 69 years (range, 58–77) for patients with localized PCa, 71 years (range, 76–81) for patients with locally advanced disease, and 71.5 years (range, 53–87) for patients with metastatic PCa. The control group had a median age of 67 years (range, 58–83).

**Table 1:** Baseline demographics and clinicopathological parameters of the study subjects.

	*localized*	*advanced*	*metastatic*	*control*
**Patients (n)**	18	7	18	11
**Median age, y (range)**	**69** (58–77)	**71** (76–81)	**71.5** (53–87)	**67** (58–83)
**Race, ethnicity**	Caucasian	Caucasian	Caucasian	Caucasian
**Gleason score (%)**	**2+3** (5.8)	**2+3** (14.3)		
	**3+3** (17.6)			
	**3+4** (58.8)	**3+4** (28.6)	**3+4** (35)	
	**4+3** (17.6)	**4+3** (28.6)	**4+3** (5)	
		**4+4** (28.6)	**4+4** (20)	
		**4+5** (14.3)	**4+5** (30)	
			**5+4** (10)	
			**5+5** (5)	
**Median baseline PSA ng/ml (range)**	**0.254**	**0.023**	**26.7**	**1.75**
	(0.003–10)	(0.04–4.2)	(0.04–1387)	(0.52–15.7)
**Primary therapy**				
*TURP*, *no (%)*				**11** (100)
*Surgery (RP)*, *no*. *(%)*	**16** (88.9)	**7** (100)	**2** (11.1)	
*Radiation*, *no*. *(%)*	**5** (27.8)	**3** (42.9)	**10** (55.5)	
**Systemic therapy**				
*Androgen treatment*, *no*. *(%)*	**3** (16.6)	**6** (85.7)	**18** (100)	
*Chemotherapy*, *no*. *(%)*			**13** (65)	

**Control:** volunteers with BPH, **RP:** Radical Prostatectomy, **TURP**: Transurethral Resection of the Prostate

In this study, 88.9% of patients with lPCa underwent open or laparoscopic radical prostatectomy. All locally advanced prostate cancer patients had positive surgical margins (PSM), and 85.7% of the patients received androgen treatment. All patients with metastatic PCa underwent androgen-deprivation therapy, and 65% of the patients received docetaxel first-line chemotherapy. Two patients were initially treated with radical retropubic prostatectomy.

### Detection of CTCs in different PCa stages

The aim of our proof-of-concept study was to test the functionality of the CellCollector in a preclinical step; therefore, blood samples were studied *ex vivo* and used in the fluid dynamic system, which system provided improved conditions for the *ex vivo* application of the wire. The in vivo application of the CellCollector was not possible at this time point because BfArM approval had not been obtained for this device.

**Fig 3:**
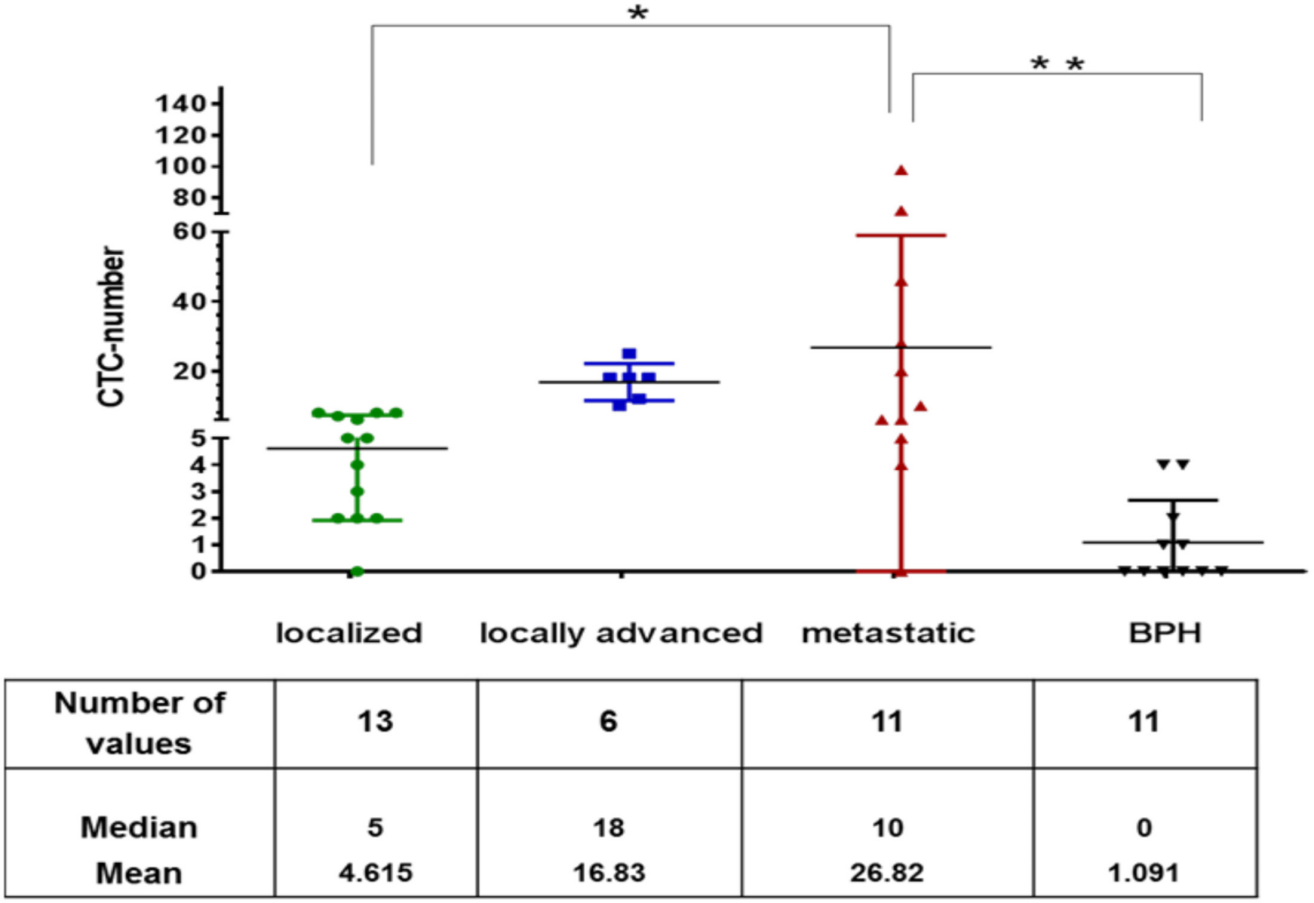
CTC counts in the study population. Each horizontal bar shows the median value. ** *p*≤ 0.01 value and * *p* ≤ 0.05.

First we evaluated the capture efficiency of the CellCollector with cultured LNCap cells. LNCap cells were spiked into blood of healthy donors. Overall, the percentage of LNCap cells detected after sample processing ranged from 35% at the higher concentration (200 cells/ml) to 10% at the lower LNCap cell concentration (~50 cells/ml). The CTC counts were analyzed in a total of 31 PCa patients and 11 control subjects. Fig 3 shows the scatterplot of all CTC counts.

The control group consisting of BPH patients had a mean of 1.09 CTCs and a median of 0 CTCs (range, 0–4), corresponding to a detection rate of 31.5%. The BPH patients underwent a transurethral resection of the prostate (TURP) to confirm the diagnosis.

Localized PCa patients displayed a mean of 4.6 CTCs and a median of 5 CTCs (range, 0–8), with a detection rate of 92.3%. These two groups showed no statistically significant difference *(p = 0*.*615)* in CTC counts.

Of the 7 patients diagnosed with laPCa, we detected a mean of 16.83 CTCs and a median of 18 CTCs (range, 10–25), with a 100% detection rate. Of these patients, 85.7% underwent androgen treatment, and 100% underwent a radical prostatectomy. The laPCa patients displayed a non-significant difference *(p = 0*.*39)* in CTC count compared to the localized PCa group. The CTC number was clearly higher, as in the PCa-l group.

**Fig 4:**
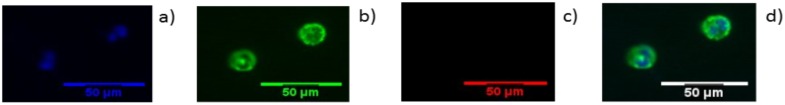
Immunocytochemical analysis of CTCs captured with the CellCollector^™^ in the blood of prostate cancer patients. The CTCs were identified and enumerated via a) positive nuclear staining (Hoechst), b) positive cytokeratin staining, and c) negative CD45 staining. d) Overlay of all images showing size and morphological characteristics.

Among the patients with metastatic progression, we detected a median of 10 CTCs and a mean of 26.83 CTCs (range, 0–98), with a 90.9% detection rate. The CTC count of mPCa patients was significantly different than that of patients with localized PCa *(p = 0*.*01)* and the control group *(p = 0*.*0068)*. Nonetheless, there were no significant differences *(p = 0*.*55*) in the CTC counts between the two groups of advanced PCa patients. An important finding of our pilot study was that phenotyping the CTCs on the CellCollector was very user-friendly. The immunocytochemical analysis of the wire demonstrated a clear surface with low contamination by non-specific binding, cell debris and artifacts (Fig 4). The binding of EpCAM-positive leucocytes was very constant in a range of 5 to 10 per wire resulting in a relation of captured CTCs to leukocytes of 50% (5 leucocytes and 5 CTCs) in localized PCa and about 94.6% (5 leucocytes and 89 CTCs) in metastatic PCa patients.

### Identification of cancer-specific transcripts in captured CTCs

Initially, we investigated the possibility of performing an mRNA analysis on LNCap cells captured by the wire (50, 200 or 500 cells spiked per 15 ml of blood). In the LNCap cells captured by the CellCollector, the mRNA levels of EGFR, PSA and PSMA were detectable in all spiked samples. All analyzed markers and the housekeeping gene actin showed greater mRNA levels as the concentration of added cells increased. The concentrations of cancer-specific transcripts varied, with 0.31 ng/μl of PSMA in 50 spiked LNCap cells and 35.72 ng/μl of PSA in 500 spiked LNCap cells (Table 2).

**Table 2:** Multiplex RT-PCR performed after isolating spiked LNCap cells.

LNCaP Cell Counts	Actin-Signal [ng/μl]	EGFR-Signal [ng/μl]	PSMA-Signal [ng/μl]	PSA-Signal [ng/μl]
**50**	6.7	2.76	0.31	30.62
**200**	9.56	4.94	0.66	31.46
**500**	12.41	13.17	3.21	35.72

Next, the molecular profiling of captured CTCs was performed in 5 blood samples obtained from lPCa patients and 7 blood samples obtained from mPCa patients (Table 3). We were able to detect cancer-specific transcripts in the CTCs captured by the wire; these transcripts are described in Table 3. The transcripts were identified only in 4 out of 12 (33.3%) blood samples obtained from PCa patients. The classification of blood samples showed that 4 (57.1%) of the blood samples from mPCa patients (n = 7), were positive for one cancer-specific transcript. The EGFR transcript was identified in 3 patients (42.8%) undergoing systemic therapy for metastatic cancer. The PSMA transcript was detected in one patient (14.3%) undergoing androgen-deprivation therapy. The whole blood samples of localized PCa patients were negative for the analyzed cancer-specific transcripts. Thus, mRNA analysis of CTCs captured by the wire is possible.

**Table 3:** Detection of CTCs by multiplex RT-PCR performed after *ex vivo* CTC isolation by the CellCollector and patient characteristics.

Patient No. & stage	Age	PSA (ng/ml)	Gleason	Primary therapy	Systemic therapy	Positive transcripts in CTCs
**1 l**	**77**	**0.05**	**4+3**	**2, 3**	**1**	**Neg.**
**2 m**	**75**	**0.06**	**4+5**	**2**	**1, 2**	**EGFR**
**3 m**	**68**	**7.9**	**4+5**	**1, 3**	**1, 2**	**Neg.**
**4 l**	**73**	**8.5**	**4+3**	**2**		**Neg.**
**5 m**	**87**	**82.5**	**4+4**	**3**	**1**	**Neg.**
**6 m**	**55**	**27.3**	**3+4**	**3**	**1**	**Neg.**
**7 m**	**70**	**5.1**	**4+5**	**3**	**1**	**PSMA**
**8 l**	**82**	**0.77**	**3+4**	**2**		**Neg.**
**9 l**	**53**	**6.1**	**3+4**	**2**		**Neg.**
**9 m**	**66**	**8.6**	**3+4**	**3**	**1, 2**	**EGFR**
**11 l**	**58**	**7.3**	**3+4**	**2**		**Neg.**
**12 m**	**78**	**44.6**	**5+5**	**1, 2, 3**	**1**	**EGFR**

**l:** localized PCa, **m:** metastatic PCa, **Primary therapy;** TURP [1], Surgery (RP RP = Radical Prostatectomy) [2], Radiation [3], ** Systemic therapy:** Androgen treatment [1], Chemotherapy [2]

### CTC number and clinical outcomes

**Fig 5:**
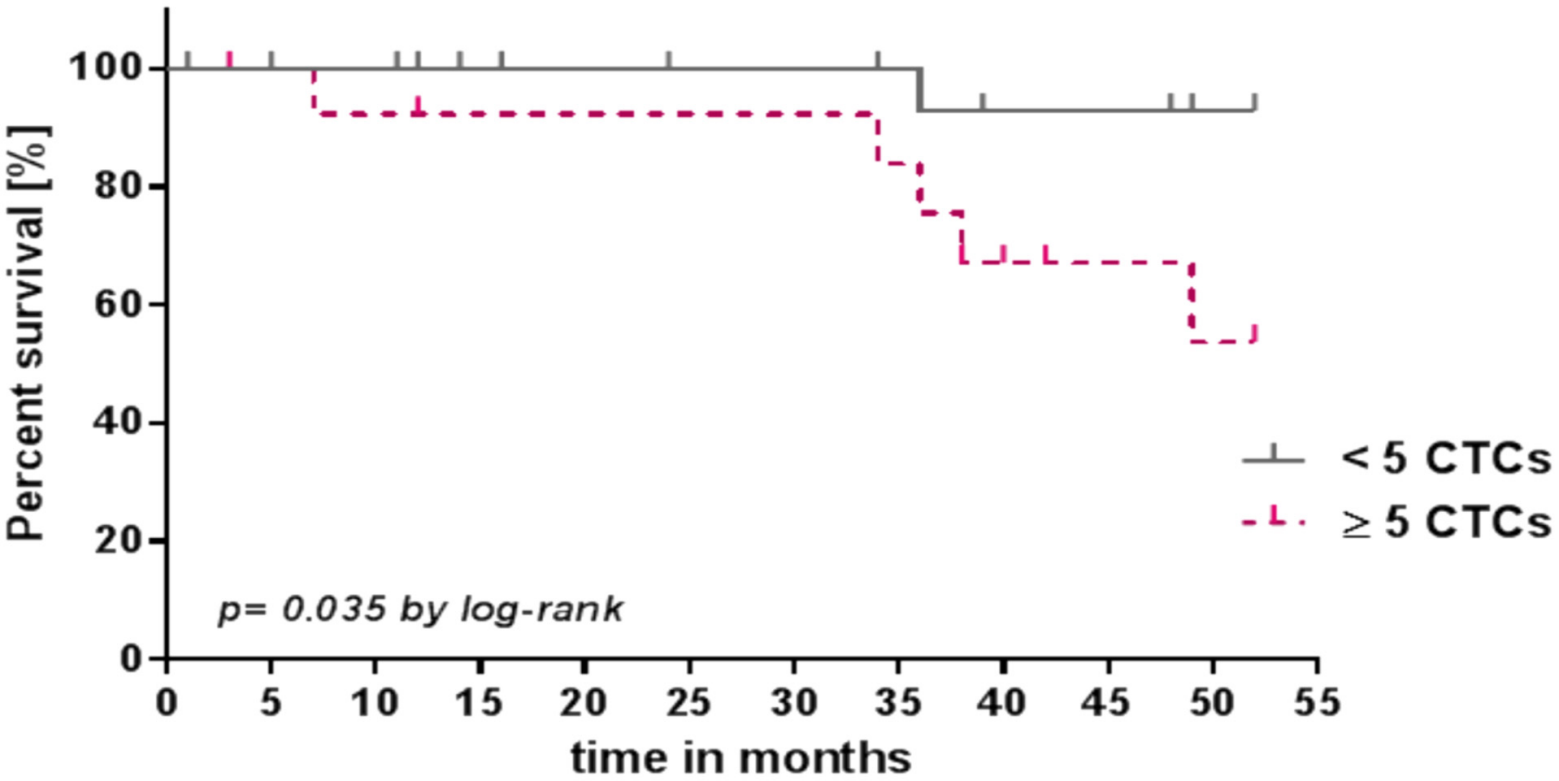
Survival rates (4.5 years) of patients with CTC counts < 5 CTCs and ≥ 5 CTCs. The median survival times are not defined.

For the survival analysis, based on the literature, we used a cut-off point of ≥ 5 CTCs to divide the patients into two groups independent of cancer stage. In the present proof of concept, the Kaplan-Meier analyses demonstrated that patients with >5 CTCs had a mortality risk that was 7.0 times greater than that of patients with ≤5 CTCs (hazard ratio (HR) 7.0, 95% confidence interval: 1.1–29.39). There was a significant difference *(p = 0*.*035)* in the overall survival (OS) between the two groups. The median OS was undefined for both groups (Fig 5).

## Discussion

CTCs have the potential to provide timely information about disease progression and may help to fill the gap between diagnosis and therapy monitoring. Furthermore, CTCs provide the ability to take a “liquid biopsy” and represent a noninvasive tool for obtaining tumor samples at different time points, thereby allowing physicians to validate therapeutic decisions and obtain actual information about the cancer’s phenotype [5,13,14].

Our proof-of-concept study reports a new functionalized medical wire (CellCollector) intended for the *in vivo*-and *ex vivo-*isolation of CTCs [9]. The first step in this study was the preliminary application of the wire to blood samples *ex vivo*. This preclinical step was required for the approval of the application of CellCollector *in vivo* by the Federal Institute for Drugs and Medical Devices (BfArM). The wire captured CTCs expressing the cell surface marker EpCAM, which is the same criterion approved by the FDA for the use of the CellSearchSystem in isolating CTCs. We also used the FDA-approved criteria for the identification of CTCs [2]. This definition of CTCs did not allow cancer-specific identification. Our findings first demonstrated that it is possible to use the wire to isolate CTCs *ex vivo* from patients with different stages of PCa. We detected mean numbers of CTCs in the blood of BPH and local PCa patients who were below the 5-CTC threshold. We found that 92.3% of the local PCa group (n = 12) was positive for CTCs. We expect that CTCs are extremely rare and are thus unlikely to be detected in localized PCa patients. The low number of CTCs in BPH and local PCa patients did not allow any disease-specific conclusion. Furthermore, the follow up in the two groups demonstrated that the CTC count had no effect on the survival of local PCa and BPH patients. Therefore, the results of the CellCollector study suggest that a CTC number above or below a disease-specific cut off value is required. A combination of the counts and the genetic characterization of captured cells are urgently needed to achieve better clinical usefulness for CTC parameters for clinicians and patients. Previous studies have identified CTCs in localized PCa patients. Moreno and colleges used FACSCalibur and CellQuest to calculate a mean value of 5.0 CTCs in 7-ml blood samples from lPCa patients [15]. In another pilot study, CTCs were captured using CTC-Chip (anti-EpCAM) and detected in 42% of PCa patients before surgical tumor removal [16]. We confirmed these results, although our results showed no significant differences between the control group and lPCa patients. For this reason, it is necessary to design an additional step for the PCa-specific characterization of the CTCs. This step should induce additional prognostic and therapeutic target information about the captured cells [17,18]. Antonarakis et al. demonstrated that the androgen-receptor splice variant 7 (AR-V7) messenger RNA in CTCs from patients with advanced PCa may be associated with resistance to enzalutamide and abiraterone [19]. These results suggest the benefits and usefulness of CTCs in the clinical setting. Our metastatic cancer patients displayed a median of 10 CTCs and a mean of 26.8 CTCs. Interestingly, all patients had undergone systemic therapy (100% androgen treatment and 65% chemotherapy) at the time of blood sampling. This could indicate that the cancer was progressing during treatment. In the mPCa group, relating the CTC count with clinical parameters demonstrated only a moderate correlation with the serum PSA level (r = 0.345) and the Gleason sum (r = 0.222). The other study groups showed no correlations between the CTC count and the clinical or histological parameters. Previous studies have also revealed no or only moderate correlations between the CTC count and other known prognostic parameters [20,21]. In a previous study, the IMMC38 trail confirmed that the PSA titers from patients with progressive, metastatic, castration-resistant PCa were either weakly correlated or not correlated with survival. Those authors summarized that lactate dehydrogenase (LDH) and CTC counts were strongly correlated with survival time [22]. A critical step in CTC isolation in metastatic PCa patients is the epithelial-mesenchymal transition (EMT), which is associated with downregulation of EpCAM expression on the CTC surface. The reduced expression of epithelial markers might therefore result in false negative results [23].

However, we were able to distinguish between the patients’ survival prognoses based on their CTC counts. Patients with ≥ 5 CTCs had significantly shorter survival times (*p* = *0*.*035*) independent of the baseline characteristics of the groups. Our preliminary findings confirmed that CTCs are an independent prognostic marker for OS.

Prior studies have verified that CTC enumeration can be used to predict prognoses in patients with metastatic, castration-resistant PCa [2,3,21,22,24]. The largest data sets were obtained with the CellSearchSystem, which has received FDA clearance and undergone broad clinical validation [1,2,21]. However, a limitation of the CellSearchSystem is its required blood volume, and thus, this platform has low sensitivity [25]. Another limitation is the low purity of the isolated cells [8]. Therefore, molecular characterization of the captured CTCs is difficult with this system [26–28]. The clean surface of the CellCollector used in our pilot study constitutes a considerable advantage for the immunocytochemical characterization of captured cells. As a result, immunocytochemical characterization and enumeration are user friendly. Additionally, fewer leukocytes, artifacts and cell debris are captured by the wire surface. Our results show that the molecular phenotyping of PCa-CTCs captured by the wire is possible (Table 3). Initially, we used spiking experiments to check the wire’s sensitivity. It is possible to molecularly characterize captured cells, and the spiked cell counts represent the marker concentrations of the cell counts. However, it can be challenging to spike the correct cell count into the blood samples because this technique requires experience in cell counting under a microscope.

Finally, we achieved a sensitivity of 57.1% in the mPCa group and negative results in the lPCa group. We identified the cancer specific transcripts EGFR and PSMA in CTCs. Only one of the 7 patients had decidable PSMA expression. This result suggests that CTCs may have a heterogeneous phenotype. PSMA expression is known to increase in higher-grade PCas, during cancer progression, and following castration. Another advantage of the PSMA protein is its stable expression during the EMT [8,28]. Gleghorn et al. developed a device that allowed for immunocapturing by the PSMA antibody. The geometrically enhanced differential immunocapture (GEDI) microfluidic device captured a median of 54 CTCs from the blood of castration-resistant PCa patients undergoing taxane chemotherapy [29,30].

The EGFR signal in the captured CTCs is not specific for prostate cancer, but this factor plays a central role in cell proliferation, migration, motility, invasion, and survival in normal and malignant cells. Among our analyzed blood samples of PCa-m (n = 7), 42.8% (n = 4) were positive for EGFR mRNA in CTCs. These results suggest cancer progression and might be a valuable tool in the near future. The limited results from the molecular analysis of captured CTCs may confirm the tumor heterogeneity, which has been described in detail in epithelial cancer types [20]. These types of analyses are very important because, if specific markers are used, it is feasible to identify which CTCs have aggressive or dormant behaviors. Currently, various different platforms exist for CTC isolation. However, challenges remain, including the need for a robust technology to detect CTCs with high sensitivity and specificity. This technique should reflect the rarity, fragility and heterogeneous phenotypes of the CTCs. Furthermore, we must specifically characterize PCa CTCs to provide more information in addition to the CTC count.

One limitation of our pilot study is, of course, the small study population. However, the 55-month patient follow-up was sufficient to demonstrate the prognostic value of our method. The CellCollector is a CTC-detection system that allows for the molecular characterization of CTCs on the mRNA level and through the immunocytochemical analysis of the captured CTCs.

## Conclusion

Our data demonstrated that at different stages of PCa, the sensitive isolation and molecular characterization of CTCs *ex vivo* by the CellCollector are feasible. This proof of concept was required to obtain BfArM approval of the CellCollector in a clinical study for the *in vivo* isolation of CTCs in the blood stream of PCa patients. Clearly, larger prospective trials using the CellCollector are needed to evaluate the method. This exploratory study reveals the opportunity to apply a CTC-isolation technique that allows for the counting and molecular characterization of CTCs.
